# Evaluating the cost of NP-led vs. GP-led primary care in British Columbia

**DOI:** 10.1177/08404704241229075

**Published:** 2024-01-30

**Authors:** Damien Contandriopoulos, Katherine Bertoni, Rita McCracken, Lindsay Hedden, Ruth Lavergne, Gurprit K. Randhawa

**Affiliations:** 1 175083 University of Victoria, Victoria, British Columbia, Canada.; 2 12358 University of British Columbia, Vancouver, British Columbia, Canada.; 3 1763 Simon Fraser University, Burnaby, British Columbia, Canada.; 4 3688 Dalhousie University, Halifax, Nova Scotia, Canada.

## Abstract

In 2020, British Columbia (BC) opened four pilot Nurse Practitioner Primary Care Clinics (NP-PCCs) to improve primary care access. The aim of this economic evaluation is to compare the average cost of care provided by Nurse Practitioners (NPs) working in BC’s NP-PCCs to what it would have cost the government to have physicians provide equivalent care. Comparisons were made to both the Fee-For-Service (FFS) model and BC’s new Longitudinal Family Physician (LFP) model. The analyses relied on administrative data, mostly from the Medical Services Plan (MSP) and Chronic Disease Registry (CDR) via BC’s Health Data Platform. Results show the cost of NPs providing care in the NP-PCCs is slightly lower than what it would cost to provide similar care in medical clinics staffed by physicians paid through the LFP model. This suggests that the NP-PCC model is an efficient approach to increase accessibility to primary care services in BC and should be considered for expansion across the province.

## Introduction

The lack of timely access to primary care has been a significant problem across Canada for decades. Nearly 1 in 7 Canadians reported not having a regular healthcare provider in 2019^1^ and, in 2020, 40% reported having an avoidable emergency department visit due to the lack of primary care options.^2^ The situation has also significantly worsened since the COVID-19 pandemic.^3,4^ At the time of writing, almost one million British Columbians do not have a Primary Care Provider (PCP).^5^

Increased involvement of non-physicians such as Registered Nurses (RNs) and Nurse Practitioners (NPs) has long been recognized as an effective way to enhance access to primary care services.^6-12^ In Canada, NPs are RNs with substantial clinical experience and additional specialized master’s or doctorate-level training. NPs have a broader scope of practice compared to RNs, including the ability to independently diagnose, prescribe treatments and tests, manage chronic conditions, and refer patients to specialist physicians.

Various models have been employed to integrate NPs into the delivery of primary care services. Among these models, the NP-led clinic stands out for its potential to disrupt the traditional healthcare landscape.^13^ In these clinics, there is no family doctor on-site and NPs assume the role of lead professionals within an interdisciplinary team, providing a comprehensive range of primary care services to a dedicated patient panel.^6,14^

In 2020, British Columbia (BC) opened four pilot NP-led clinics.^15^ The BC model was named Nurse Practitioner Primary Care Clinic (NP-PCC). The pilot NP-PCCs are located on Vancouver Island (Victoria, Nanaimo, and Qualicum Beach) and one is on the mainland (Surrey). These clinics have been tasked with delivering comprehensive primary care services to residents from formally defined catchment areas who lack a primary care provider. The clinics receive block funding from the British Columbia Ministry of Health but operate under the governance of a self-appointed board of directors with a significant degree of managerial autonomy.

Each clinic has an enrolment target of 6,800 patients within the initial three years of their operation. Patients are formally registered with the clinic as well as also being informally connected to a specific NP within the clinic. Each clinic is funded to have a staff of six full-time NPs, one of whom also holds the role of clinic director, a dedicated social worker, a mental health counsellor or clinician, two RNs, and four medical office assistants.^16,17^

Many studies with strong research designs have shown nursing-intensive primary care models to be safe and effective.^8,11,18-26^ Measurable clinical outcomes are systematically found to be equivalent^11,18,19,23,27-32^ to those produced by medical models, though patient satisfaction tends to be higher for nurse-provided services.^11,19,21,26,27,29,30,33,34^ There also are some robust studies comparing the cost of care provided by nurses—generally NPs—to the cost of care provided by physicians. This literature generally reports similar^27,34-37^ or marginally lower^29,38-41^ costs for nurse-led services. Nevertheless, the economic impact of nurse-led primary care remains less documented than its clinical quality.^9,20,23,26,42-44^ It is also important to note that cost-effectiveness results are fully dependent on the relative compensation levels for physicians and nurses in the jurisdiction where the data is collected.

## Objectives

The aim of this economic evaluation is to benchmark the average cost of NP-PCC care compared to longitudinal physician-based models from BC.

## Methods

### Study design

The study design rests on a convergent parallel mixed-method approach. The main component is a descriptive quantitative analysis of administrative data. Additional context to better understand actual practice and therefore more accurate billing comparison was developed via qualitative interviews conducted with NPs and clinic directors.

### Quantitative data description

The quantitative data used here was accessed through two platforms: The Health Data Platform British Columbia (HDP-BC) and Health Data Coalition (HDC) Discover. HDP-BC is a secure, cloud-based data research and analysis platform provided by the Ministry of Health to support research and analysis within the health sector and in academia. HDC Discover is a web-based data analytics tool that connects data from various electronic medical record systems in primary care across BC. In HDP-BC, data from the Medical Services Plan (MSP) and Chronic Disease Registry (CDR) data holdings were extracted for the four pilot NP-PCCs for the 2022 calendar year using SQL queries. Aggregated data on disease prevalence for all 4 NP-PCCs were accessed using HDC Discover. The NP-PCC budgets from the MOH’s clinic funding letters were used as the basis for actual costs of NP care in NP-PCCs.^16^ Both the NPs’ salaries and the cost of overheads were included in the computations.

### Qualitative data

The benchmarking algorithms and final cost estimates are informed by interview-based qualitative data collected from clinic directors and NPs working in the NP-PCC model. NPs were recruited through the NP-PCCs to participate in 30-60 minute interviews that were transcribed using the transcription feature in Zoom Cloud. Transcriptions were reviewed and revised for accuracy using the recordings.

### Quantitative data analysis

To compare the actual average cost of NP-PCC care to what it would have cost the province to have that care provided in physician-based models, two different benchmarks were developed. The first benchmark is based on the cost of physicians working in BC’s Fee-For-Service (FFS) model and the second one is based on the province’s new Longitudinal Family Physician (LFP) payment model. Both benchmarks are based on re-coding NP encounter codes into comparison billing that would approximate MSP’s physician payment schedules. NP encounter codes are non-billable codes used to report on services provided to patients.

Until early 2023, physicians providing primary care services in BC were mostly paid through FFS.^45^ In Canada, what is described as FFS is generally a blended model that combines FFS per se with other approaches such as pay for performance, incentives, etc.^46-48^ Physicians typically bill one service for each visit, and in some specific situations more than one. Physicians can also bill yearly bonuses if they perform preventative care or if they provide complex clinical care, such as chronic disease management.

At the end of 2022, BC announced the launch of a new compensation model for primary care physicians providing longitudinal care (LFP model). The LFP model is also a blended compensation model and consists of three parts: (1) a per visit fee, (2) an hourly rate that can be billed for both direct (time spent in patient visits) and indirect patient care (for example, time spent reviewing imaging results and completing paperwork), and (3) a quarterly payment based on the complexity of a physician’s panel.^49^ However, at the time of conducting the analyses, only the first two components were implemented. While the LFP model is still in its implementation phase, it has already had a large uptake from physicians.^50^

It is also important to note that, as it is the case for in most practice settings in Canada, in both the FFS and LFP models, family physicians have to fund their office overhead out of their billings. Overheads vary from one practice setting to the next but, on average, amount to almost a third of total billings for family physician in Canada.^51^ Although there are some funding avenues to help support clinic operating costs,^52,53^ there is no published evaluation about the extent of business costs these funding supports actually cover.

The benchmarking algorithms developed here were programmed to turn any of the 153 NP encounter codes identified in the dataset into equivalent physician billing codes and amounts. Many of the comparison billing algorithms also relied on patients’ age and some on aggregated volume and cost from average medical practice.^54^ For the FFS benchmark, a different set of comparison billings were also produced for physician bonuses (e.g., chronic disease management incentives) billable for longitudinal care. The LFP payment model benchmark relied on the number of hours worked by NPs and a simplified per-visit amount.

## Results

### Patient complexity in NP-PCCs

Comparing the four NP-PCCs to the average of all primary care clinics in BC reveal a higher average prevalence of several health conditions, especially mental health, mental health and substance use, chronic pain, neurodevelopmental disorders, asthma, musculoskeletal disorders, and sexually transmitted diseases. However, the prevalence is slightly lower compared to average for chronic obstructive pulmonary disease, chronic kidney disease, dementia, diabetes, frailty, heart failure, hypertension, and ischaemic heart disease.

### Fee-for-service benchmark

The type of NP services outputted by the algorithm was compared with actula medical practice^54^ and found to be quite similar. By far, the bulk of NP practice in NP-PCCs is made of what would be billed as “visits,” “complete examinations,” and “consultations” in MSP’s FFS structure.

The FFS algorithm produced an average dummy billing cost per service of $41.66. The FFS benchmarking total is $3,478,844, whereas the NP-related budget for the NP-PCCs is $5,640,000. As such, the FFS benchmarking (Table 1) corresponds to approximately 62% of the NP-related budget (compensation and overheads) for the NP-PCCs.

**Table 1.** Final results of fee-for-service benchmarking.

Clinics	Billable visit-related amount	Nb of services	Average cost per service	Total bonus-related amount	Total FFS benchmark	NP-related NP-PCC budget
Clinic 1	$568,770	11,301	$50.33	$418,150	$986,920	$1,410,000
Clinic 2	$413,468	11,304	$36.58	$423,604	$837,072	$1,410,000
Clinic 3	$305,079	8,289	$36.81	$310,684	$615,764	$1,410,000
Clinic 4	$518,121	12,448	$41.62	$520,965	$1,039,086	$1,410,000
**Grand total**	**$1,805,440**	**43,342**	**$41.66**	**$1,673,403**	**$3,478,844**	**$5,640,000**

### LFP payment model benchmark

The LFP payment model benchmarking is based on the two components that had been implemented at the time of conducting the analyses: the hours worked and a simplified per-visit amount. Table 2 below provides a summary of the results. The total time-based amount was $5,241,600 and the volume-based amount was $1,140,115, totalling $6,381,715. Given the NP-related budget for the NP-PCCs was $5,640,000, the LFP payment model benchmarking reflects savings of $741,715 or 13% of the NP-related budget for the NP-PCCs.

**Table 2.** Summary of LFP payment model benchmarking.

Clinics	Visit-based amounts	Time-based amounts	Total LFP benchmark	Savings or costs	NP-related NP-PCC budget
Clinic 1	$288,140	$1,293,617	$1,581,757	$171,757	$1,410,000
Clinic 2	$290,290	$1,355,128	$1,645,418	$235,418	$1,410,000
Clinic 3	$225,680	$1,015,201	$1,240,881	($169,118)	$1,410,000
Clinic 4	$336,005	$1,577,652	$1,913,657	$503,657	$1,410,000
**Grand total**	**$1,140,115**	**$5,241,600**	**$6,381,715**	**$741,715**	**$5,640,000**

## Discussion

Our results suggest that the relative affordability of NPs services is highly dependent on the benchmark used (i.e., FFS or LFP model). For multiple reasons discussed below, the authors believe the LFP benchmark provides both a more relevant and a more appropriate reference point.

### Fee-for-service benchmarking

First, it is important to stress that the FFS benchmarking has several limitations. NP interviews revealed that in day-to-day practice, there are potentially billable visits that are not currently being reported in NP encounter codes. The interviews suggested there was only limited training and resources available to support encounter coding for NPs, especially after recent changes to the NP encounter codes. This translated into a high variability in NPs’ confidence, comfort, knowledge, understanding, and skills in encounter coding. It also appears that many NPs do not follow up on encounter codes that end up rejected by the billing submission system. Given NPs are paid on a contractual basis, the reporting incentive is very different compared to physicians paid through the FFS or LFP models, where their income directly depends on it. It is, therefore, likely that the benchmarking underestimates the volume and total cost of the services provided by the NP-PCCs.

### Volume of care

In the same way, there is a documented tendency of FFS to artificially increase the volume of care by rewarding multiple short visits (one issue per visit)^48^ instead of having fewer and more comprehensive visits when dealing with complex patients. Some clinics have even implemented a “one issue per visit” policy.^55,56^ However, such volume-based incentives do not exist for NPs in the NP-PCC model. Thirdly, an analysis of the HDC Discover data and NP interviews suggest a high level of medical and psychosocial complexity for patients who were rostered and cared for by the NP-PCC clinics. The FFS algorithm was not adjusted to address these limitations. However, if adjustments were made to account for 30% underreporting of visits in encounter codes and 40% to account for differences in the way the clinical practice is organized, the cost of NP-PCCs would be roughly equivalent to FFS medical settings. For the reasons discussed above and given the LFP payment model is becoming the de-facto dominant model for the provision of longitudinal care in BC, the authors believe it constitutes a better reference point.

### Operational capacity

It is also important to stress that the four NP-PCCs opened with small teams at various time points in 2020. Over time, the clinic teams have grown in size and extent of services. Given this economic evaluation focuses on 2022 data, it should be emphasized that the NP-PCCs had not yet reached their full operational capacity. Research shows it takes physicians over 20 years in practice to reach their peak productivity.^57^ It is thus likely that the volume of care provided by the NP-PCCs will significantly increase in the coming years. Any increase in the volume of care provided by the NP-PCCs would further improve the efficiency of the model relative to medical clinics.

Overall, the study results suggest that the reliance on NP-PCCs to provide comprehensive longitudinal care to unattached patients likely constitutes a cost-effective and efficient approach.

## Lessons for health leaders

In most jurisdictions, primary care delivery systems are struggling to respond to the needs and expectation of the population. This situation has multiple causes but all solutions have in common the need to provide more and better primary care with limited resources (people, time, equipment, and knowledge^58^). This economic evaluation provides health leaders with evidence about the relative cost of an NP-led model of care that was otherwise shown to produce desirable clinical results.^59^ As such, the results can help decision-makers to better understand the potential of alternative care delivery models to address the current primary care crisis. This study also puts forward some policy-level considerations (for example, the time required for new teams to achieve peak productivity or the low external validity of cost-effectiveness studies) that should be taken into account to optimize the impact, scalability, and sustainability of new models of care.

## Limitations

This economic evaluation was conducted in the context of new clinics opening during the height of the COVID-19 pandemic. Almost all the patients were previously unattached, which created a substantial level of extra work for initial visits and onboarding that is not reflected in visit counts. Additionally, as previously discussed, the comparison of costs for NP-led vs. physician-led care are highly dependent on the level and mode of compensation of both professional groups. Further, the present study does not have a control group and cannot account for the relative cost-effectiveness of the different types of professionals.

## Conclusion

The results obtained in this economic evaluation found potential costs savings when comparing the cost of care provided by NPs to the cost BC would have incurred if the same care had been provided by physicians. The study results also suggest that the relative affordability of NPs’ services is highly dependent on the benchmark used. For the reasons discussed earlier, the costs comparison with the LFP payment model appears to be more reliable. It is also now the dominant model for the provision of longitudinal care in BC and thus the de-facto reference.

These results suggest that the NP-PCC model is a cost-effective and efficient approach to increase accessibility to primary care services in BC. Further, parallel research focused on the socio-demographic profile of patients and their care experience before joining the NP-PCCs^60^ as well as research on patients’ health and care experience in NP-PCCs^59^ suggest that the NP-PCC model also constitutes an effective way to address current problems in accessing good quality longitudinal care in BC. The authors believe the results support exploring the expansion of the NP-PCC model beyond the four pilot sites studied in British Columbia.
